# Problem Gambling and Delinquent Behaviours Among Adolescents: A Scoping Review

**DOI:** 10.1007/s10899-018-9754-2

**Published:** 2018-02-22

**Authors:** David T. Kryszajtys, Tara E. Hahmann, Andrée Schuler, Sarah Hamilton-Wright, Carolyn P. Ziegler, Flora I. Matheson

**Affiliations:** 1 Dalla Lana School of Public Health, University of Toronto, 155 College St, Toronto, ON M5T 3M7 Canada; 2 Centre for Urban Health Solutions, St. Michael’s Hospital, 30 Bond St, Toronto, ON M5T 3M7 Canada; 3 Department of Family and Community Medicine, Centre for Urban Health Solutions, St. Michael’s Hospital, 30 Bond St, Toronto, ON M5B 1W8 Canada; 4 Health Sciences Library, St. Michael’s Hospital, 30 Bond St, Toronto, ON M5B 1W8 Canada; 5 Centre for Criminology and Sociolegal Studies, University of Toronto, 14 Queen’s Park Cres W, Toronto, ON M5S 3K9 Canada; 6 Mental Health and Addictions Research Program, Institute for Clinical Evaluative Studies, G1 06, 2075 Bayview Avenue, Toronto, ON M4N 3M5 Canada

**Keywords:** Problem gambling, Delinquency, Public health, Adolescents, Scoping review

## Abstract

Despite many studies indicating an association between problem gambling and delinquent behaviours among adolescents, there has been no effort to systematically analyze the state of the literature on this relationship. To fill this gap, we conducted a scoping review of the literature published between 2000 and 2016 on problem gambling and delinquent behaviours among adolescents. We searched twelve databases and reviewed reference lists to identify eligible studies. Search terms included a combination of medical subject headings and keywords for gambling, youth, and delinquency, which were combined with the Boolean operator “AND”. 1795 studies were identified through the literature search. Nine studies were eligible for inclusion. All of the studies were conducted in North America, with primarily male participants, and most of the data were cross-sectional. No qualitative studies met the inclusion criteria. Screening tools used to measure problem gambling were inconsistent, making comparisons across studies difficult. We found a consistent moderate to strong association between problem gambling and delinquent behaviour. Only one study presented associations by socio-economic status and none considered gender, sex or ethnic differences. Studies in the review showed that problem gambling is associated with both violent and non-violent behaviours among adolescents. These associations may suggest that problem gambling and delinquent behaviours have common risk factors and reflect a syndrome of risky behaviours best targeted through prevention and treatment that is holistic and considers the context in which the youth is situated. Further research is warranted to better understand the relationship between problem gambling and delinquent behaviours.

**Electronic supplementary material:**

The online version of this article (10.1007/s10899-018-9754-2) contains supplementary material, which is available to authorized users.

## Introduction

### Adolescent Problem Gambling: A Public Health Issue

Adolescent gambling has emerged as a serious public health concern as the use of emerging gambling technologies (e.g., free social media gambling) increase the likelihood that adolescents—under 19 years of age—will gamble for money (Gainsbury et al. 2016; Meerkamper 2010). Parents and teachers are unaware of and deprioritize gambling as a public health issue for adolescents in comparison to other risky behaviours (Campbell et al. 2011; Derevensky 2012; Derevensky et al. 2014). However, gambling is associated with multiple harms among adolescents that include legal harms, harms to family, finances, employment, and mental and physical health (Shaffer and Korn 2002; Lambie and Randell 2013). Along with these harms, one of the more serious and less studied harms of adolescent gambling, the development of delinquent behaviours, can lead to involvement with the criminal justice system (Magoon et al. 2005). These harms make problem gambling a key area of concern for the public health community (Shaffer and Korn 2002). Improved understanding of the relationship between problem gambling and delinquent behaviours among adolescents may reduce the public health harms associated with them.

### Prevalence of Problem Gambling Among Adolescents

Available systematic reviews of the prevalence of problem gambling among adolescents indicate that rates across studies and countries range from 0.2 to 12.3% (Calado et al. 2016; Volberg et al. 2010). Rates of adolescent at-risk gambling range from 10 to 15%, and rates of adolescent engagement in some form of gambling activity range from 60 to 80% (Calado et al. 2016; Volberg et al. 2010). These differences in prevalence may relate to inconsistencies in screening and measurement of adolescent gambling across studies and countries (Derevensky et al. 2003; Shaffer and Korn 2002). There is agreement among researchers, however, that adolescent rates of gambling participation and problematic gambling exceed those of adults (Gupta and Derevensky 2000; Molinaro et al. 2014; Volberg et al. 2010). Considering the high rates of gambling in this population, adolescence marks a critical period for gambling prevention and intervention (Magoon et al. 2005).

### Problem Gambling and Delinquent Behaviours

In the most recent review on the topic to date, Magoon et al. (2005) reported on criminal acts associated with gambling. They reported on two studies that found higher rates of problem and pathological gambling among incarcerated (21%) in comparison to non-incarcerated adolescents, although as suggested by other research problem gambling can precede delinquent behaviours. For example, adolescents may steal to fund their gambling activities. They also found studies reporting associations between gambling and truancy, selling drugs, shoplifting, stealing money, and working for ‘bookmakers’. Overall, their research found that adolescents who gamble are more likely to participate in or have a history of committing delinquent acts, especially if they gamble at a problem or pathological level. Furthermore, the frequency and amount of money spent on gambling activities is a significant predictor of delinquent activities. Several gaps in the knowledge on this topic were identified: the influence of the parent/child interaction on development of problem gambling and delinquent behaviours, the types of gambling associated with delinquency, and how theft within and outside of the home is related to the progression of both problem gambling and delinquent behaviours.

The documented link between problem gambling and delinquent behaviours among adolescents warrants a review of the current state of the literature to see how research has progressed and where gaps remain. The main objective of this scoping review was to identify the available evidence on the association between adolescent delinquent behaviour and problem gambling. A secondary objective is to identify measures of problem gambling and delinquent behaviours in the literature.

## Methods

The recommendations of Arksey and O’Malley (2005) form the basis of the methodology for this scoping review. They suggest a five-stage approach: (1) identify the research question, (2) identify relevant studies, (3) select relevant studies, (4) chart the data, (5) collate, summarize and report the results.

### Search Strategy

In June 2016, the following databases were searched: OVID PsycINFO, Ovid MEDLINE, CINAHL, Criminal Justice Abstracts, Child Development and Adolescent Studies; Social Sciences Abstracts, Applied Social Sciences Index and Abstracts (ASSIA), International Bibliography of the Social Sciences (IBSS), ProQuest Dissertations and Theses Global, Social Services Abstracts, Sociological Abstracts, Web of Science: Social Science Citation Index. We selected these databases to ensure interdisciplinary coverage, including social sciences, criminal justice, child and adolescent studies, medicine, allied health professions, nursing, and psychology. The search terms included a combination of medical subject headings and keywords for the concepts of gambling, youth, and delinquency, combined with the Boolean operator “AND”. The search strategy was developed by an information specialist with input from the project team (please see Appendix A for the full MEDLINE strategy). We included English language scholarly articles published between January 1st, 2000 and June 16th, 2016.

### Study Selection

**Fig. 1:**
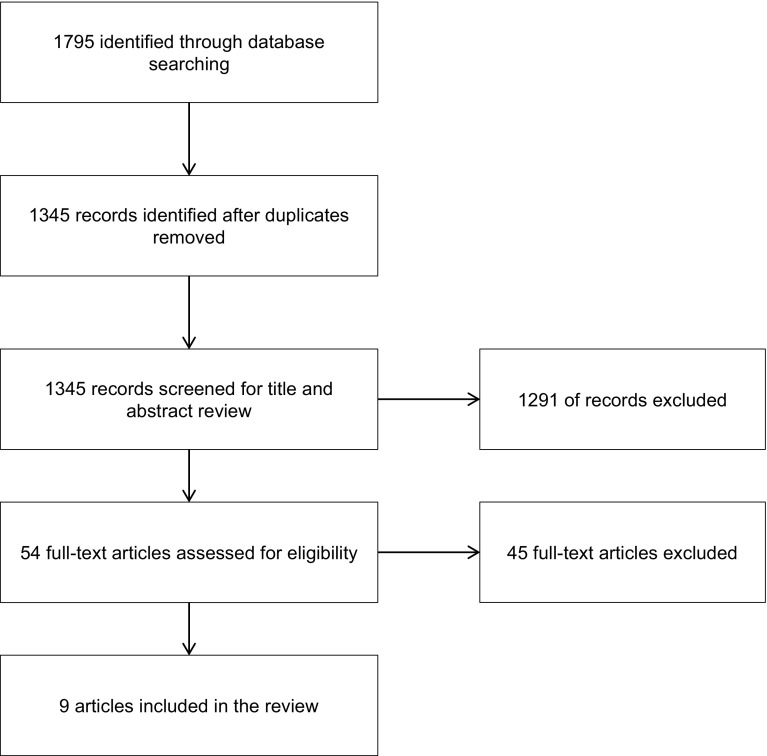
Flow diagram of study selection

Our research objectives and our initial review of the literature informed the inclusion and exclusion criteria for this scoping review. Studies were accepted if the sample included adolescents under the age of 19 and if the study examined the relationship between problem gambling and delinquent behaviours. Only studies using a validated diagnostic tool or screening instrument for problem or pathological gambling were included. For the purposes of this review, we defined delinquent behaviours as those that result in or are likely to result in direct physical or financial harm to others and are illegal. We excluded substance use, truancy, and underage drinking to focus our definition of delinquency that would otherwise encompass a broad spectrum of behaviours (Cox and Allen 2007). Examples of delinquent behaviours within this definition include violence, theft, dealing drugs, carrying weapons and driving in excess of the speed limit. Conduct disorder is also included, as it includes delinquent behaviours within its definition—a persistent display of serious antisocial actions that are extreme for the child’s developmental level and impinge on the rights of others (American Psychiatric Association 2013). Study designs that meet the inclusion criteria include randomized controlled trials, observational studies (cohort, cross-sectional, case–control), descriptive, qualitative, and mixed methods studies. Study selection followed a multi-step process. First, three team members independently reviewed ten studies to pilot eligibility criteria for the abstract and title review. The same three team members refined the inclusion/exclusion criteria based on the pilot and then independently reviewed titles and abstracts of all studies identified through the search strategy. Figure 1, the study selection flow chart, shows that 1795 studies were identified through the literature search with duplicates removed. There was an agreement on inclusion and exclusion for 94% of the studies at this stage. When necessary, a fourth team member helped resolve conflicts. Fifty-four studies were included in the full-text review. To pilot the full-text review, two team members reviewed five studies. These team members independently reviewed fifty-four full-text studies for eligibility. We accepted nine articles for final review and data extraction with a 92.6% agreement rate between two reviewers, drawing on a third team member to help resolve conflicts.

### Data Extraction

For eligible publications, two team members independently extracted information using a data extraction tool the team developed, piloted, and modified. Extraction items included publication details (authors and dates), theory, research objectives, research design, sample demographics, information on tools used to measure problem gambling and delinquency measures, and findings on the relationship between problem gambling and delinquency (both significant and non-significant). We report odds when available; otherwise, group differences in proportions, means, or correlations are reported. A third team member verified that the data that was extracted accurately reflected how it was reported in the eligible publications.

## Results

### Sample Description

Table 1 shows descriptive information about each study. Of the nine studies included in this review, all were conducted in North America; five were conducted in the United States and four in Canada. The average age of participants across studies ranged from 13 to 19. The majority of studies included both males and females (n = 7). Two studies focused on exclusively male samples (n = 2). Of the nine studies deemed eligible for review, four studies examined student populations, two studies examined a non-specific adolescent population, one study examined an adolescent population experiencing incarceration, and an additional study examined an adolescent patient population. Three studies examined socio-economic status, focusing primarily on low-income status or participants in a disadvantaged neighborhood. Eight of the studies were cross-sectional and one utilized longitudinal data (see Table 1). No qualitative studies met the inclusion criteria.

**Table 1:** Descriptive information (sample size, age, sex, race/ethnicity/socio-economic status (SES)/types of participants/location/types of studies) of studies meeting criteria for scoping review

Author	Sample size	Age	Sex	Race/ethnicity	SES	Type of participants	Location	Type of study
Wanner et al. (2009)	Sample 1, n = 502; Sample 2, n = 663)	Mean = 16.2 SD = 0.5–0.6	100% male	100% Caucasian	Sample 1: low SES (participants from economically disadvantaged areas in Quebec), sample 2: middle-class^a^	Students	Canada	Longitudinal^b^
Cook et al. (2015)	n = 4851	Mean = 14.6	47% male, 53% female	N/A	N/A	Students	Canada	Cross-sectional
Goldstein et al. (2013)	n = 249	Mean = 16.9 SD = 1.3	30.1% female, 69.9% male	59.4% African American; 30.9% Caucasian; 9.7% Asian, American Indian	Mostly low-SES (58.7% of sample reported their family received public assistance)	Emergency department patients	U.S.A	Cross-sectional
Husted, et al. (2006)	n = 1051 n = 562 (relevant subsample: youth of driving age)	Range = 13–17	52.1% male, 47.9% female	76.9% Caucasian, 6.8% African American, 10.1% Hispanic, 6.1% Native American/Asian/Other	N/A	Residents^c^	U.S.A	Cross-Sectional
Magoon et al. (2007)	n = 55	Mean = 15.3, SD = 1.49	73%male, 27% female	N/A	N/A	Adolescents in Detention Centers	Canada	Cross-sectional
Slavin et al. (2013)	n = 2276	Range = 14–18	56% male,44% female	N/A	N/A	Students	U.S.A	Cross-sectional
Vitaro et al. (2001)	n = 717	Range = 16–17	100% male	100% Caucasian	Low SES (participants form disadvantaged neighborhood in Montreal)	Students	Canada	Longitudinal
Welte et al. (2009)	n = 2,274	Range = 14–21 Range 14–19 (relevant subsample)	Mix (distribution N/A)	N/A	N/A	Residents	U.S.A	Cross-sectional
Potenza et al. (2011)	n = 2006	Range = 14–18	61% male, 39% female	N/A	N/A	Students	U.S.A	Cross-sectional

^a^Participant’s parents answered the Blishen et al. (1987) Occupational Prestige Scale: an SES index that classifies occupations according to income and education in Canada. A mean score for both parents was computed

^b^Only cross-sectional data was extracted from this study due to our inclusion and exclusion criteria

^c^Study recruited adolescents by calling homes and obtaining consent from parents to interview adolescent participant

### Problem Gambling Screening Tools and Measures

See Table 2.

**Table 2:** Descriptions of screening tools and measures used to assess problem gambling and delinquent behaviours, and the types of delinquent behaviours examined using these tools for studies meeting scoping review criteria

Author	Problem gambling screening tools or measures (including prevalence)	Delinquent behaviour screening tools or measures	Types of delinquent behaviour constructs examined using delinquent behaviour screening tools or measures^a^
Wanner et al. (2009)	South Oaks Gambling Screen, Revised Adolescent Version (French)—Examines gambling problem severity score (12/12 items) Gambling Severity (score from 1 to 12) Sample 1 (M = 1.50, SD = 1.30) Sample 2 (M = 0.41, SD = 1.21)	Diagnostic Interview Schedule for Children and Self-Reported Delinquency Questionnaire	Theft, Violence
Cook et al. (2015)	South Oaks Gambling Screen, Revised Adolescent Version (Partial—6/12 items) (Short scale—which may be overly inclusive in the PG category) 2.8% PG	Self-Report Questions: from the (Ontario Student Drug Use and Health Survey Delinquency Measure: Any individual who endorsed 3 or more of any of the nonviolent or violent delinquency items from the Ontario Student Drug Use and Health Survey was classed as delinquent.	Non-violent crime: Theft under $50; Theft over $50; Vandalism; Break and Enter; Take car without consent; Sell marijuana or hashish; sell drugs other than marijuana; fire setting; Violent crime: Assault; Gang fight; carry weapon; carry handgun;
Goldstein et al. (2013)	South Oaks Gambling Screen, Revised Adolescent Version (Partial 5/12 items) Low Consequence Gambler = 62.24%; High consequence Gamblers = 38.8%	Peer violence by Add Health Survey and Conflict Tactics Survey; Dating violence by Conflict in Adolescents Dating Relationships Inventory	Peer violence, Dating violence
Husted, et al. (2006)	The Diagnostic Statistical Manual IV (All items) 4.3% problem gambling.	Question about exceeding speed limit	High-Risk Speeding
Magoon et al. (2007)	The Diagnostic Statistical Manual IV-Juvenile (All Items) 18% Probable Pathological gamblers	The Diagnostic Statistical Manual IV-Juvenile and Gambling Questionnaire	Delinquent acts committed in order to gamble: Taken money from someone else live with; illegal acts; stolen money; stolen money from outside the family or shoplifted;
Slavin et al. (2013)	Massachusetts Gambling Screen (12/40 items) 27% At-Risk/Problem Gambling	Self Report Question: Fighting, from Youth Behavior Risk Survey Carrying a weapon (past month)	Fight involvement, Carrying a weapon.
Vitaro et al. (2001)	South Oaks Gambling Screen, Revised Adolescent Version (French) (12/12 items) Severity Score Gambling severity (1–12) Age 16 (M 0.36, SD = 0.23) Age 17 (M 1.13, SD = 2.4)	Self-Reported Delinquency Questionnaire (27 items, 4 points per item)	Delinquency
Welte et al. (2009)	South Oaks Gambling Screen, Revised Adolescent Version (12/12 items) 6.5% At-Risk/Problem Gambling	National Institute for Mental Health Diagnostic Interview for Conduct disorder; Diagnostic Interview Schedule for Children	Conduct disorder
Potenza et al. (2011)	Massachusetts Gambling Screen (12/40) 34% At-risk/Problem Gambling	Self-Report Question: Getting into a serious fight.	Getting into serious fights; carrying a weapon;

^a^Delinquent behaviour constructs are only included in this list if they are also associated with problem gambling, as per our inclusion criteria

### Screening Tools Based on the South Oaks Gambling Screen-Revised Adolescent

Table 2 provides information on the screening tools and measures of problem gambling and delinquency used in the nine selected studies. Five out of nine studies used the South Oaks Gambling Screen-Revised Adolescent (SOGS-RA: Winters et al. 1993) (Cook et al. 2015; Goldstein et al. 2013; Vitaro et al. 2001; Wanner et al. 2009; Welte et al. 2009). The SOGS-RA is a screening tool that assesses gambling severity in adolescents over the past 12 months and is comprised of twelve items that are related to the DSM-IV (American Psychiatric Association 1994) criteria for pathological gambling. Items are answered either yes (scored 1) or no (scored 0) and higher scores indicate more problems related to gambling. A score of two or three is considered at-risk gambling and a score of four or more is considered problem gambling.

Vitaro et al. (2001) and Wanner et al. (2009) used the French version of the SOGS-RA. Cook et al. (2015) used a modified version of the SOG-RA, containing six out of twelve items, embedded in the Ontario Student Drug Use and Health Survey data (OSDUHS, a cross-sectional survey of Ontario students enrolled in grades seven through twelve: Boak et al. 2014). Goldstein et al. (2013) used a five-item modified version of the SOGS-RA further defining two sub-classes of participants who gamble: low-consequence gamblers (LCG) and high consequence gamblers (HCG). Participants in the HCG’s group were more likely to score a “yes” on any of the five SOGS-RA items than those in the LCG group. Using the SOGS-RA, Welte et al. (2009) combined at-risk and problem gambling into current at-risk/problem gambling.

### Screening Tools Based on the Diagnostic Statistical Manual

Slavin et al. (2013) and Potenza et al. (2011) used twelve items from the Massachusetts Gambling Screen (MAGS: Shaffer et al. 1994) that reflect the ten DSM-IV inclusion criteria for pathological gambling (Potenza et al. 2011; Slavin et al. 2013). Low-risk gambling was defined as having participated in gambling activity in the past year but having no positive responses to the twelve items on the MAGS. A participant who reported one or more DSM-IV criteria was classified as an at-risk/problem gambler.

Husted et al. (2006) used the ten DSM-IV inclusion criteria to create categories of at-risk gambling and problem gambling, citing this as a commonly employed strategy in other prevalence studies of problem gambling. At-risk gambling was defined as endorsing one to two of these criteria. Problem gambling was defined as endorsing three to four criteria and pathological gambling as endorsing five or more. Magoon et al. (2007) used The Diagnostic Statistical Manual IV-Juvenile (DSM-IV-J) which was developed specifically for adolescents. It contains nine dimensions comprised of twelve items based on the criteria for pathological gambling (American Psychiatric Association 1994). Each dimension is scored either one (yes) or zero (no). A score of four or greater is considered pathological gambling.

### Delinquency Screening Tools and Measures

Two studies used the Self-Reported Delinquency Questionnaire (SRDQ: Blanc and Frechette 2013) (Vitaro et al. 2001; Wanner et al. 2009). The SRDQ examines involvement in delinquent behaviours over the last 12 months and is comprised of twenty-seven items. It includes three subscales; a physical violence scale, theft scale, and vandalism scale.

Two studies used the Diagnostic Interview Schedule for Children (DISC: Shaffer et al. 1993) (Wanner et al. 2009; Welte et al. 2009). Wanner et al. (2009) drew from the DISC for the assessment of theft and violence in the previous 12 months. Welte et al. (2009) also used the DISC, specifically the section which operationalizes DSM-IV criteria for conduct disorder. The questions in this section reflect delinquent behaviours and include questions about fighting, bullying, carrying dangerous weapons, and theft in the last 12 months.

For measures of violence, Goldstein et al. (2013) used the Add Health Survey (Sieving et al. 2001). Items included fights with friends and strangers. The study also used the Conflict in Adolescent Dating Relationships Inventory (Wolfe et al. 2001) to measure interpersonal conflict through the occurrence of fights in dating relationships.

Four studies (Cook et al. 2015; Husted et al. 2006; Potenza et al. 2011; Slavin et al. 2013) did not use pre-developed screening tools for delinquent behaviours, but, instead, either borrowed self-report questions from another survey or developed their own questions to assess delinquent behaviours within the last year. Slavin et al. (2013) used “past-month” and “past-year” as a time-line in their question of weapon possession.

Five studies described violent delinquent behaviours (Cook et al. 2015; Goldstein et al. 2013; Potenza et al. 2011; Slavin et al. 2013; Wanner et al. 2009). Within the violence category, five studies described physically violent behaviours against others, including measures such as assault, fighting, violence, and fire setting (Cook et al. 2015; Goldstein et al. 2013; Potenza et al. 2011; Slavin et al. 2013; Wanner et al. 2009). Three studies described carrying a weapon (Cook et al. 2015; Potenza et al. 2011; Slavin et al. 2013).

Four studies described non-violent delinquent behaviours (Cook et al. 2015; Husted et al. 2006; Magoon et al. 2007; Wanner et al. 2009), three of which described various types of theft (e.g., taking a car without consent, taking money from people outside of the family, etc.) (Cook et al. 2015; Magoon et al. 2007; Wanner et al. 2009). Other non-violent behaviours included selling drugs, vandalism (Cook et al. 2015), high-risk speeding (Husted et al. 2006), and illegal acts (Magoon et al. 2007).

Three studies used measures that assessed the presence or absence of one or more delinquent behaviours (e.g., measures of delinquency or conduct disorder) (Cook et al. 2015; Vitaro et al. 2001; Welte et al. 2009).

### Associations Between Problem Gambling and Delinquency

See Table 3.

**Table 3:** Statistical associations between delinquent behaviours and problem gambling for studies meeting scoping review criteria

Study	Delinquent behaviour	Statistical association	*p* value	Comparison
Violent				
Cook et al. (2015)	Assault	OR 7.5; PG (47.4%) non-PG (9%)	*p* < 0.01; CI, 3.5–16.3	PG versus non-PG
Cook et al. (2015)	Carry a handgun	OR 11.2; PG (14.4%) non-PG (1.1%)	*p* < 0.01; CI 3.8–33.0	PG versus non-PG
Cook et al. (2015)	Carry a weapon	OR 4.8; PG (31.8%) non-PG (6.8%)	*p* < 0.01; CI 2.4–9.6	PG versus non-PG
Slavin et al. (2013)	Carry a weapon (only participants who report fighting) (past month)	OR 16.5	CI 3.85–70.69	ARPG versus NG
Slavin et al. (2013)	Carry a weapon (only participants do not report fighting) (past month)	OR 3.21	CI 2.09–4.95	ARPG versus NG
Potenza et al. (2011)	Carry a weapon (only participants who do not report gambling on the internet)	OR 1.90	*p* < 0.001	ARPG versus LRG
Potenza et al. (2011)	Carry a weapon (only participants who report gambling on the internet)	OR 2.11	*p* < 0.005	ARPG versus LRG
Slavin et al. (2013)	Fighting	NG (6.73%), LRG, (38.57%), APRG (54.71%)	*p* < 0.0001	NG, LRG, APRG
Cook et al. (2015)	Fighting (Gang)	OR 11.3; PG (23.8%), non-PG (2.3%)	*p* < 0.01; CI 5.0–25.2	PG versus non-PG
Potenza et al. (2011)	Fighting (serious) (only participants who do not report gambling on the internet)	OR 1.93	*p* < 0.005	ARPG versus LRG
Potenza et al. (2011)	Fighting (serious) (only participants who report gambling on the internet)	OR 2.50	*p* < 0.005	ARPG versus LRG
Cook et al. (2015)	Fire setting	OR 3.4; PG (41.8%), non-PG (14.1%)	*p* < 0.01; CI 1.9–6.2	PG versus non-PG
Wanner et al. (2009)	Violence	r = 0.25 (sample A) r = 0.16 (sample B)	*p* < 0.05	Gambling Severity Score
Goldstein et al. (2013)	Violence (Dating)	(Mean, SD) LCG (2.4, 3.4) HCG (4.2, 4.6)	*p* < 0.01	LCG versus HCG
Goldstein et al. (2013)	Violence (Peer)	(Mean, SD) LCG (9.4, 8.6) HCG (16.1, 11.6)	*p* < 0.01	LCG versus HCG
Non-violent				
Cook et al. (2015)	Break and enter	OR 6.1, non-PG (4%) PG (24.7%)	*p* < 0.01; CI 3.4–11.0	PG versus non-PG
Husted et al. (2006)	High-risk speeding	NG (14%), LRG (26%), ARG (50%), PG (70%)	*p* < 0.0001	NG, LRG, ARG, PG
Magoon et al. (2007)	Illegal acts	Non-PPG (11.1%), PPG (60%)	*p* < 0.01	Non-PPG versus PPG
Cook et al. (2015)	Sell drugs other than Marijuana	OR 19.6, PG (29.2%) non-PG (1.5%)	*p* < 0.01; CI 10.4–36.9	PG versus non-PG
Cook et al. (2015)	Sell Marijuana or Hashish	OR 5.3, PG (31.1%) non-PG (5.9%)	*p* < 0.01; CI 2.9–9.5	PG versus non-PG
Wanner et al. (2009)	Theft	r = 0.22 (sample A) r = 0.14 (sample B)	*p* < 0.05	Gambling Severity Score
Cook et al. (2015)	Theft (over $50)	OR 14.5, PG (44.5%) non-PG (4.3%)	*p* < 0.01; CI 7.9–26.6	PG versus non-PG
Magoon et al. (2007)	Theft (Stolen money from outside the family or shoplifted)	Non-PPG (11.1%), PPG (40%)	*p* < 0.05	Non-PPG versus PPG
Magoon et al. (2007)	Theft (Stolen money)	Non-PPG (19.4%), PPG (60%)	*p* < 0.05	Non-PPG versus PPG
Cook et al. (2015)	Theft (Taken car without consent)	OR 8.2, non-PG (29.4%) PG (41.7%)	*p* < 0.01; CI 3.9–17.2	PG versus non-PG
Magoon et al. (2007)	Theft (Taken money from someone else they live with)	Non-PPG (11.1%), PPG (80%)	*p* < 0.0001	Non-PPG versus PPG
Cook et al. (2015)	Theft (under $50)	OR 14.5, non-PG (13.4%) PG (51.4%)	*p* < 0.01; CI 3.1–9.8	PG versus non-PG
Cook et al. (2015)	Vandalism	OR 6.8, non-PG (12.7%) PG (53.7%)	*p* < 0.01; CI 3.9–11.9	PG versus non-PG
Delinquency overall				
Welte et al. (2009)	Conduct disorder	Non-conduct disorder, PG (1.7%), non-conduct disorder, ARPG (5.2%); current conduct disorder, PG (6.1%), current conduct disorder ARPG (22.9%)	N/A	PG, ARPG
Welte et al. (2009)	Conduct disorder (for each additional increase in criteria) (participants ages 14–15)	OR = 1.8	*p* < 0.001; CI 1.4–2.2	ARPG
Welte et al. (2009)	Conduct disorder (for each additional increase in criteria) (participants ages 16–17)	OR = 1.5	*p* < 0.001; CI 1.3–1.8	ARPG
Welte et al. (2009)	Conduct disorder (for each additional increase in criteria) (participants ages 18–19)	OR = 1.3	*p* < 0.001; CI 1.1–1.6	ARPG
Cook et al. (2015)	Delinquency	OR 5.86 (in multivariate mode with substance use and internalizing problems); r = 0.24	*p* < 0.001; *p* < 0.001	PG versus non-PG; PG
Vitaro et al. (2001)	Delinquency (Delinquency at 17 and PG at 16)	r = 0.19	*p* < 0.01	PG severity score
Vitaro et al. (2001)	Delinquency (PG and delinquency at 16)	r = 0.29	*p* < 0.01	PG severity score
Vitaro et al. (2001)	Delinquency (PG at 17 and Delinquency at 16)	r = 0.22	*p* < 0.01	PG severity score
Vitaro et al. (2001)	Delinquency (PG at 17 and Delinquency at 17, controlling for Peer deviancy, impulsivity, and parental supervision at ages 13–14	r = 0.22	*p* < 0.001	PG severity score

*PG* problem gambling, *PPG* pathological problem gambling, *ARG* at-risk gambling, *ARPG* at-risk problem gambling, *LRG* low risk problem gambling, *LCG* low consequence gambling, *HCG* high consequence gambling, *NG* no gambling

### Violent Delinquent Behaviours

Cook et al. (2015) reported higher odds of violent behaviour among respondents experiencing problem gambling relative to those not involved in problem gambling. The behaviours examined included assault (OR 7.5, CI 3.5–16.3), fire setting (OR 3.4, CI 1.9–6.2), gang fights (OR 11.3, CI 5.0–25.2), carrying a weapon (OR 4.8, CI 2.4–9.6), and carrying a hand gun (OR 11.2, CI 3.8–33.0).Slavin et al. (2013) found that 6.73% in the non-gambling group, 38.57% in the low-risk gambling group, and 54.71% in at-risk/problem gambling group reported fighting (*p* < 0.0001). Te odds of carrying a weapon were higher among at-risk gambling/problem gambling respondents relative to those who did not gamble. Respondents experiencing at-risk/problem gambling were more likely to carry a weapon in the past month regardless of whether they were involved (OR 16.5, CI 3.85–70.69) or not involved in fights (OR 3.21, CI 2.09–4.95). No significant interactions between fighting and non-fighting respondents experiencing problem gambling were observed.

Goldstein et al. (2013) examined mean differences in peer and dating violence among low consequence gamblers (LCG) and high consequence gamblers (HCG). Peer violence was higher in the HCG group ($\bar{x}$ = 16.1, SD = 11.6) than the LCG group ($\bar{x}$ = 9.4, SD = 8.6) (*p* < 0.01) as was dating violence in the HCG group in comparison to the LCG group ($\bar{x}$ = 4.2, SD = 4.6; $\bar{x}$ = 2.4, SD = 3.4; *p* < 0.01).

Potenza et al. (2011) explored the association between gambling, fighting and carrying a weapon among internet and non-internet gamblers. Respondents who placed bets on the internet and experienced at-risk/problem gambling were at higher risk of involvement in serious fights (OR 2.50, *p* < 0.005) and carrying a weapon (OR 2.11, *p* < 0.005) relative to those who gambled on the internet but were categorized as low-risk. Similarly, respondents who did not participate in internet gambling but who experienced at-risk/problem gambling were more likely to report serious fights (OR 1.93, *p* < 0.005) and carrying a weapon (OR 1.9, *p* < 0.0001) relative to those at the lower risk threshold of problem gambling. The authors found no significant interaction between internet and non-internet gambling and gambling group (at-risk/problem vs. low-risk). Wanner et al. (2009) reported a strong correlation between violence and gambling problems among low-SES adolescents (r = 0.42, *p* < 0.05) and a moderate correlation among middle-class adolescents (r = 0.16, *p* < 0.05).

### Non-Violent Delinquent Behaviours

Cook et al. (2015) found that theft was higher in the problem gambling group than the non-problem gambling group, and this included theft under $50 (OR 5.5, CI 3.1–9.8, *p* < 0.01), theft over $50 (OR 14.5, CI 7.9–26.6, *p* < 0.01), breaking and entering (OR 6.1, CI 3.4–11.0), and taking a car without consent (OR 8.2, CI 3.9–17.2, *p* < 0.01). Similarly, respondents experiencing problem gambling had a higher likelihood of involvement in vandalism (OR 6.8, CI 3.9–11.9, *p* < 0.01), selling marijuana or hashish (OR 5.3, CI 2.9–9.5, *p* < 0.01), and selling drugs other than marijuana (OR 19.6, CI 10.4–36.9, *p* < 0.01) than respondents not experiencing problem gambling. Wanner et al. (2009) reported a moderate correlation between gambling problems and theft (r = 0.34; *p* < 0.05) among their low-SES and middle-class samples (r = 0.14; *p* < 0.05).

Magoon et al. (2007) found that theft was higher among respondents reporting problem gambling in comparison to adolescents in the non-problem gambling group. Of the respondents experiencing problem gambling, 80% (*p* < 0.0001) reported taking money from someone else they lived with, 40% (*p* < 0.05), reported stealing money from outside the family or shoplifting, and 60% (*p* < 0.05) reported stealing money from any source including from outside the family. For respondents who did not experience problem gambling, these rates were lower at 11.1, 11.1, and 19.4% respectively. Magoon et al. (2007) also reported a higher percentage of illegal acts to support gambling among respondents experiencing problem gambling (60%) versus respondents who were not experiencing problem gambling (11.1%; *p* < 0.01).

Husted et al. (2006) reported that respondents who experienced problem gambling had higher rates of high-risk speeding (number of times on average the participants went 10 m/h over the speed limit) than other respondents: problem gambling (70%), at-risk gambling (50%), low-risk gambling (26%), and no-gambling (14%; *p* < 0.0001).

### Nonspecific Delinquency

Several studies calculated an overall measure of delinquency to explore its relationship with gambling among adolescents. Cook et al. (2015) defined delinquency as a score of three or more on a list of twelve violent and non-violent behaviours. They reported a moderate correlation between problem gambling and delinquency (r = 0.24, *p* < 0.001).

Goldstein et al. (2013) defined delinquency as the number of delinquent behaviours reported in the past 12 months with possible scores from one to eleven. They found a higher mean number of delinquent behaviours among the high consequence gambling group ($\bar{x}$ = 8.2, SD = 8.2) compared to the low consequence group ($\bar{x}$ = 3.7, SD = 4.7).

Welte et al. (2009) defined conduct disorder as having three or more symptoms on the National Institute for Mental Health Diagnostic Interview Schedule for Children. They found that the relationship between problem gambling and conduct disorder was strong among younger age groups. For example, risk of problem gambling increased by 80% for each additional conduct disorder criteria among 14–15 year olds (OR = 1.8, CI 1.4–2.2). The odds decreased across the age groups 16–17 (OR = 1.5, CI 1.3–1.8) and 18–19 (OR = 1.3, CI 1.1–1.6). Among adolescents with conduct disorder, the rate of current problem gambling was 6.1, and 22.9% among those in the at-risk/problem gambling group. Among respondents without conduct disorder the rate of at-risk/problem gambling was lower in comparison to those with conduct disorder (1.7% for current problem gambling; 5.2% for current at-risk/problem gambling).

Vitaro et al. (2001) defined delinquency using the Self-Reported Delinquency Scale Questionnaire which includes three subscales reflecting violence, theft, and vandalism. In an initial correlation matrix, they showed that problem gambling at age 16 was correlated with delinquency at age 17 (r = 0.19, *p* ≤ 0.01) and that delinquency at age 16 was correlated with problem gambling at age 17 (r = 0.22, *p* ≤ 0.01). Using structural equation modeling that accounted for peer deviancy, parental supervision, and impulsivity at age 13, they further explored the relationship between delinquency and problem gambling at age 16 and delinquency and problem gambling at age 17. This exploration found that none of the path coefficients were significant.

## Discussion

This review examined the strength and nature of the association between problem gambling and delinquent behaviours among adolescents. We identified nine studies that were conducted between the years 2000 and 2016. The findings of our review suggest a moderate to strong association between adolescent problem gambling and other delinquent behaviours. One of the prevailing explanations for this association is that people engage in financially motivated delinquency to fund their gambling (Blaszczynski and Silove 1996; Dickerson 1989; Magoon et al. 2005). Research conducted with the adult population suggests that problem gambling is primarily associated with non-violent criminal behaviours and used to pay off debts and/or to sustain gambling (McCorkle 2002). Yet, the findings of this review documented a relationship between both non-violent and violent behaviours and problem gambling among adolescents. Only one study delved deeper to examine motivations for delinquent behaviour and found that adolescents who experienced problem and pathological gambling reported higher involvement in illegal acts, in most cases theft, and in some cases undefined illegal acts, to support their gambling activities than typical adolescents (Magoon et al. 2005).

It is clear from the findings of this review that adolescents engage in a range of delinquent behaviours, as well as gambling. Whether adolescents become involved in delinquent behaviours specifically to support gambling behaviour is not clear from our findings, especially since fire setting and high-risk speeding, on the surface, appear devoid of financial benefit. The association between problem gambling and violent acts opens the debate to other possible explanations for the association between these two social problems (Blaszczynski and Silove 1996; Dickerson 1989; Magoon et al. 2005). Findings from several studies suggest that non-violent and violent delinquent behaviours share common risk factors with problem gambling among adolescents; such as impulsivity, avoidance coping, low parental supervision, and deviant peers (Cooper et al. 2003; Dickson et al. 2006; Vitaro et al. 2001). The noted associations between problem gambling and delinquency in this review may partially reflect the over-representation of male adolescents in the studies. The association between problem gambling and delinquent behaviours among female adolescents has yet to be explored.

Jessor and Jessor (1977) developed a conceptual model of adolescent risk behaviours. They argued that risk behaviours do not emerge out of disorder; rather adolescent behaviour, whether problematic or not, emerges out of the organized social ecology of adolescent life. Adolescents’ decisions to engage in risk behaviours reflect rational thought; there are socially organized opportunities to co-learn risk behaviours. Within their framework, both the antecedents of risk behaviours and protective factors can be found in five interconnected domains: biology/genetics, social environment, perceived environment, personality, and behaviour. What emerges from their conceptualization is a complex view of human behaviour influenced by the interactions between the five domains. They argue that ‘being at risk’ relates to the development stage in the ontology of risk. For adolescents already engaged in risk behaviours the question is how engrained is the risk behaviour and what protective factors exist that promote resilience. At the earlier stage of the ontology of risk where such behaviours have not been initiated, what protective factors can be mobilized to reduce vulnerability to risk and what approaches mitigate risk.

**Fig. 2:**
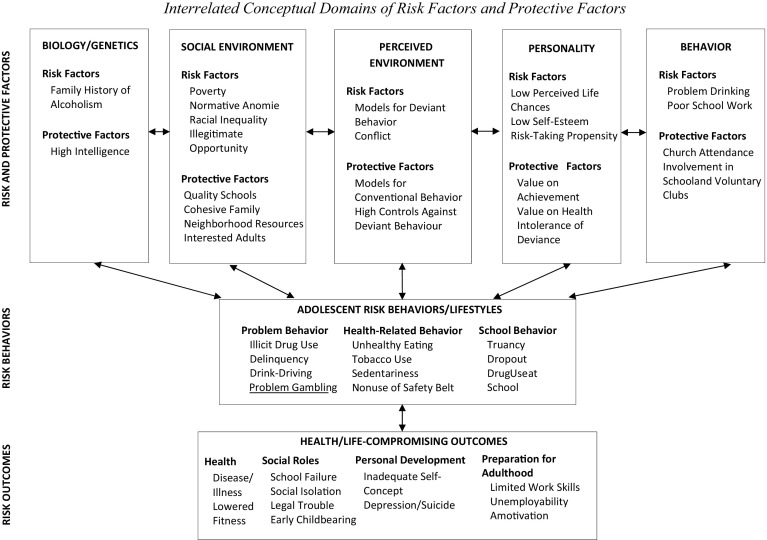
Risk and protective factors, risk behaviors and risk outcomes Adapted from: Jessor (1991, p. 602)

A recent review examined the effectiveness of addiction prevention programs for youth (Kempf et al. 2017). The authors discuss several key features within these programs that promote healthy behaviour: intense interventions over a sustained period of time; development of skills to respond to difficult situations; and, a focus on the social environment of adolescents with involvement of their adult network of parents, teachers, and social workers (Kempf et al. 2017). Early social theory argued for a bio-social-psychological approach to prevention and treatment of problematic behaviours (Ajzen 1991; Bandura 1969, 1989; Bronfenbrenner 1977; Jessor 1987, 1991; Jessor and Jessor 1977; Patterson 1975; Rosenstock 1974; Rosenstock and Strecher 1988; Sussman et al.^1995^, ^2004^). Research continues to advocate this approach, yet adolescents continue to fall through the cracks to find themselves involved in behaviours that set them on a perilous trajectory. Recent research argues that strategies which target a syndrome of risk behaviours using this more holistic approach to adolescent engagement can be effective (Cook et al. 2015; Kempf et al. 2017; Magoon et al. 2005). Instead of identifying adolescent engagement with a particular high risk behaviour, some researchers suggest assessing the magnitude of psychosocial risk across various behaviours (Jessor 1991). High risk, according to Jessor (1991) would constitute a pervasive and deep-rooted involvement in an organized pattern or lifestyle of risk behaviours alongside minimal immersion in protective behaviours in various domains including the social environment (e.g., cohesive family unit), personality (e.g., high value on academic performance), and behaviour (e.g., involvement in conventional behaviour such as extracurricular academic activities) (see Fig. 2). Treatment for adolescents involved in high risk behaviours might benefit from an approach that considers a high-risk lifestyle and lack of involvement in positive behaviours (e.g., sports, arts, spirituality) that may mitigate the impact and effects of risk factors (Pate, et al. 2000; Eccles et al. 2003; Cotton et al. 2006; Dew et al. 2008).

The secondary objective of this review was to identify how problem gambling and delinquent behaviours are defined in the literature. Measures of problem gambling were inconsistent across studies. The most widely used measure was the full version of the South Oaks Gambling Screen. Measures of delinquent behaviour were similarly inconsistent, and derived through self-report. The inconsistency in measurement makes it difficult to fully understand the nature of the relationship between both problem gambling and delinquency.

Blinn-Pike et al. (2010) summarized the research on adolescent gambling from 1985 to 2010. Their review showed that, during this time period, research was chiefly prevalence-focused, quantitative, descriptive, school-based, and atheoretical. They reported a dearth of valid and reliable screening instruments designed with child development in mind, and few studies that made use of them with racially diverse samples. The findings of our review indicate that research on adolescent gambling has not progressed substantially since the Blinn-Pike review. For instance, we highlight the lack of qualitative studies exploring the relationship between problem gambling and delinquent behaviours from the adolescent perspective. Qualitative studies can capture people’s experiences of the social conditions in which they live as Breen et al. (2013) study illustrates. In their work, adult Indigenous participants from two Australian states spoke of involvement in criminal acts linked to two drivers: family dysfunction and to support a gambling habit. Breen et al. discuss the findings in the context of colonization and discrimination and suggest that this knowledge can inform a holistic approach to rehabilitation and care for both the gambling and crime involved person and their family.

While there is a documented association between problem gambling and delinquency, we know little about how these problems co-develop, and how their co-development may differ by sex and gender, ethnicity and socio-economic status (SES). None of the nine studies examined gender, sex, or ethnicity. Only one study explored SES. Wanner et al. (2009) reported that theft and violence were more strongly associated with gambling severity among low-SES, in comparison to middle-class adolescents, but did not look at potential reasons for the differences. Longitudinal studies are needed to untangle the causal association between problem gambling and delinquency; one such as the Canadian COMPASS study which follows youth over 9 years to understand changes in youth behaviour over time (Leatherdale et al. 2014).

## Conclusion

The review has both limitations and strengths. As we included papers from January 1, 2000 to June 16, 2015, we began this review by summarizing the narrative synthesis of the literature by Magoon et al. (2005), who examined adolescent problem gambling and delinquent behaviours prior to 2000. We excluded truancy, substance abuse, underage drinking, and other criminal behaviours that do not cause direct harm to others. Doing so allowed us to focus our definition of delinquency that would otherwise include a broad spectrum of behaviours. Future research should consider an examination of other delinquent behaviours, including truancy and substance use, in relation to problem gambling.

A key finding of this review is that problem gambling is associated with both violent and non-violent behaviours and these associations are robust. This finding shifts our understanding beyond the explanation that delinquency associated with problem gambling is merely financially motivated by gambling losses. Problem gambling and delinquency may have shared risk and protective factors that reflect a syndrome of risky behaviour. As such, it will be important to consider and gather evidence on the effectiveness of more holistic approaches to treatment and prevention that target multiple risky behaviours and are sensitive to the social-structural context in which youth are situated, including socioeconomic status, gender, and race/ethnicity.

## Electronic Supplementary Material

Below is the link to the electronic supplementary material.

Supplementary material 1 (DOCX 30 kb)
