# Supplementary material for: Problem Gambling and Delinquent Behaviours Among Adolescents: A Scoping Review
Source: J Gambl Stud. 2018 Feb 22;34(3):893–914. doi: 10.1007/s10899-018-9754-2 (PMC6096515; doi:10.1007/s10899-018-9754-2)
Supplement: Supplementary file 1 — Supplementary material 1 (DOCX 30 kb) [file 10899_2018_9754_MOESM1_ESM.docx]

# Appendix A - MEDLINE Search Strategy

Ovid MEDLINE Search Strategy

Database: Epub Ahead of Print, In-Process & Other Non-Indexed Citations, Ovid MEDLINE(R) Daily and Ovid MEDLINE(R) <1946 to Present>

Search Strategy:

--------------------------------------------------------------------------------

1 Gambling/

2 (gamble* or gambling*).tw,kw.

3 betting.tw,kw.

4 casino*.tw,kw.

5 slot machine*.tw,kw.

6 or/1-5

7 Adolescent Behavior/

8 Adolescent/

9 minors/

10 exp Homeless Youth/

11 (youth* or teen* or adolescen*).tw,kw.

12 or/7-11

13 exp Crime/

14 exp Criminal Behavior/

15 exp Criminals/

16 "disruptive, impulse control, and conduct disorders"/ or firesetting behavior/

17 Conduct Disorder/

18 exp Antisocial Personality Disorder/

19 jurisprudence/ or criminal law/ or forensic psychiatry/

20 law enforcement/ or prisons/

21 Absenteeism/

22 exp Substance-Related Disorders/

23 exp Violence/

24 aggression/ or bullying/ or problem behavior/

25 alcoholics/ or drug users/ or prisoners/

26 exp Dangerous Behavior/

27 exp Alcohol Drinking/

28 Marijuana Smoking/

29 Drug-Seeking Behavior/

30 Risk-Taking/

31 (delinquen* or crime* or illegal or offense* or conduct disorder* or anti-social or antisocial or deviant or deviance or violen* or theft* or criminal justice).tw,kw.

32 or/13-31

33 6 and 12 and 32

34 Juvenile Delinquency/

35 ((high school* or junior high or school* or student* or adolescen* or young or youth* or teenager* or teen or teens or minors or boys or girls or juvenil* or underage* or under age*) adj5 (delinquen* or illegal or crime* or criminal* or offense* or offender or jail* or prison* or truancy or truant* or conduct disorder* or anti-social or antisocial or psychopath* or devian* or vandal* or violen* or theft* or shoplift* or correctional system or felon* or imprison* or incarcerat* or correctional facilit* or criminal justice system* or criminal justice setting* or corrections or correctional setting* or fighting or substance use* or substance abuse* or marijuana use* or illicit drugs or drug abuse* or arson* or gangs or sexual misconduct)).tw,kw.

36 6 and (34 or 35)

37 33 or 36

38 limit 37 to (english language and yr="2000 -Current")
